# Contribution of Open Access Databases to Intensive Care Medicine Research: Scoping Review

**DOI:** 10.2196/57263

**Published:** 2025-01-09

**Authors:** Julien Kallout, Antoine Lamer, Julien Grosjean, Gaétan Kerdelhué, Guillaume Bouzillé, Thomas Clavier, Benjamin Popoff

**Affiliations:** 1 Department of Anesthesiology and Critical Care CHU Rouen Rouen France; 2 Univ Lille, CHU Lille, ULR 2694 - METRICS: Évaluation des Technologies de Santé et des Pratiques Médicales Lille France; 3 Fédération Régionale de Recherche en Psychiatrie et Santé mentale des Hauts-de-France Saint-André-lez-Lille France; 4 Department of Biomedical Informatics CHU Rouen Rouen France; 5 LIMICS U1142, Sorbonne université & Sorbonne Paris Nord Paris France; 6 CHU Rennes, INSERM, LTSI-UMR 1099, Univ Rennes Rennes France; 7 UNIROUEN, INSERM U1096 Normandie Université Rouen France

**Keywords:** intensive care unit, ICU, big data, databases, open access, Amsterdam University Medical Centers Database, AmsterdamUMCdb, eICU Collaborative Research Database, eICU-CRD, database, screening, descriptive analysis

## Abstract

**Background:**

Intensive care units (ICUs) handle the most critical patients with a high risk of mortality. Due to those conditions, close monitoring is necessary and therefore, a large volume of data is collected. Collaborative ventures have enabled the emergence of large open access databases, leading to numerous publications in the field.

**Objective:**

The aim of this scoping review is to identify the characteristics of studies using open access intensive care databases and to describe the contribution of these studies to intensive care research.

**Methods:**

The research was conducted using 3 databases (PubMed–MEDLINE, Embase, and Web of Science) from the inception of each database to August 1, 2022. We included original articles based on 4 open databases of patients admitted to ICUs: Amsterdam University Medical Centers Database, eICU Collaborative Research Database, High time resolution ICU dataset, Medical Information Mart for Intensive Care (II to IV). A double-blinded screening for eligibility was performed, first on the title and abstract and subsequently on the full-text articles. Characteristics relating to publication journals, study design, and statistical analyses were extracted and analyzed.

**Results:**

We observed a consistent increase in the number of publications from these databases since 2016. The Medical Information Mart for Intensive Care databases were the most frequently used. The highest contributions came from China and the United States, with 689 (52.7%) and 370 (28.3%) publications respectively. The median impact factor of publications was 3.8 (IQR 2.8-5.8). Topics related to cardiovascular and infectious diseases were predominant, accounting for 333 (25.5%) and 324 (24.8%) articles, respectively. Logistic regression emerged as the most commonly used statistical model for both inference and prediction questions, featuring in 396 (55.5%) and 281 (47.5%) studies, respectively. A majority of the inference studies yielded statistically significant results (84.0%). In prediction studies, area under the curve was the most frequent performance measure, with a median value of 0.840 (IQR 0.780-0.890).

**Conclusions:**

The abundance of scientific outputs resulting from these databases, coupled with the diversity of topics addressed, highlight the importance of these databases as valuable resources for clinical research. This suggests their potential impact on clinical practice within intensive care settings. However, the quality and clinical relevance of these studies remains highly heterogeneous, with a majority of articles being published in low–impact factor journals.

## Introduction

Intensive care units (ICUs) provide care for critical patients at high risk of morbidity and mortality. These patients, due to their severity, require continuous monitoring and surveillance of clinical, biological, and imaging parameters. This generates a large amount of data, usually collected in electronic health records. Although collected for health care purposes, these data can also be used secondarily to address other objectives, which has become a key issue in recent years [1-3]. In addition to traditional epidemiological and clinical research, the secondary use of these databases allows the emergence of new research themes such as the development of diagnostic tools, decision support systems, or predictive models of therapeutic response [4,5]. However, due to the sensitive nature of health data and the technical, legal and ethical challenges, medical data are still difficult to access [6].

During the last decades, collaborative ventures have enabled the emergence of large open access databases [7]. Building on the opportunity of digital transformation, they facilitate data sharing on a large scale and enable knowledge creation more efficiently. Furthermore, they are part of an ecosystem in which science is more transparent, reproducible, and constitutes an effective lever for scientific integrity [8]. Since the 2000s, we have seen the release of several open access ICU databases [9]. The best-known example is the Medical Information Mart for Intensive Care (MIMIC) database, which integrates anonymized, comprehensive clinical data from more than 50,000 intensive care admissions from Beth Israel Deaconess Medical Center in Boston, Massachusetts [10,11]. These open databases have led to the production of numerous research works [12,13].

Currently, little effort has been made to analyze the medical literature generated from these open intensive care databases. Previous systematic reviews were interested in describing these databases to determine their exploitation potential [9] or focused on describing machine learning techniques used to train models from these databases [14]. However, none of them were interested in the research themes and their potential impact on clinical practice.

We propose a comprehensive synthesis of clinical research publications based on ICU open databases. A scoping review was used as the most suitable research methodology for mapping this research area [15]. Our objectives were to (1) examine the bibliometric characteristics and authorship patterns of these publications and (2) investigate the research themes and methodologies used. The objective was to glimpse the contribution of these open databases in intensive care clinical research.

## Methods

### Overview

The protocol adheres to the reporting guidance provided in the PRISMA-ScR (Preferred Reporting Items for Systematic Reviews and Meta-Analyses extension for Scoping Reviews) [16] (Table S1 in Multimedia Appendix 1). The design of this study is conceived according to the Joanna Briggs Institute Reviewers’ manual for evidence synthesis [17].

### Eligibility Criteria

We selected studies based on the following criteria (Textbox 1).

**Inclusion and exclusion criteria.**

**Inclusion criteria**

- They were original articles based on open databases of patients admitted to intensive care units, covering all types of interventions and outcome measures.
- They were studies with a clinical aim (diagnosis, therapeutic, prognosis), where “clinical” pertains observations made on actual patients as opposed to theoretical, laboratory, or computer-based studies.

**Exclusion criteria**

- Studies on patients admitted outside of intensive care units.
- Studies where open databases were solely used for external validation.
- Studies not written in English.
- Systematic reviews and meta-analyses.

### Search Strategy

First, the literature was searched for open access ICU databases. A recent review systematically identified publicly available adult clinical ICU databases [9]: Amsterdam University Medical Centers Database (AmsterdamUMCdb) [7], eICU Collaborative Research Database (eICU-CRD) [18], High time-resolution ICU dataset (HiRID) [19], MIMIC clinical databases version II, III, and IV [10,20,21].

We then used PubMed-Medline (US National Library of Medicine), Embase (Elsevier), and Web of science (Clarivate Analytics) to search articles published from the inception of each database to August 1, 2022. The search terms of the different databases are included in Table S2 in Multimedia Appendix 1. A senior librarian [GK] developed and validated search queries. Finally, the references of the included articles were examined for potentially relevant medical articles that may have escaped the literature search.

### Selection

Two independent reviewers, a senior intensivist [BP] and a junior intensivist [JK], conducted the search strategy and retrieved the references. An initial selection was made after examining the titles and abstracts of the medical articles resulting from the search strategy. The reviewers [BP and JK] performed an initial blind and independent selection of the articles. Subsequently, the reviewers met to reach a consensus on any disagreements. If the 2 primary reviewers could not reach a consensus, the third reviewer, a health data engineer (AL), intervened to resolve the disagreements through arbitration. The disagreements typically revolved around the eligibility criteria for study inclusion. The selection process was facilitated using the Rayyan software [22].

### Data Extraction, Collection, and Analysis

Medical articles were referenced by their Digital Object Identifier, the name of the first author, and the date of publication. Two reviewers [BP and JK] independently extracted data using a custom electronic form designed specifically for this study. The form was pilot tested with a random sample of 20 articles to ensure its effectiveness. Upon achieving consistent data abstraction (κ≥0.8) [23], reviewers proceeded with data extraction for the entirety of included articles using the Goupile software [24].

Data for journal research fields and impact factors were obtained from Web of Science [25]. In addition, research topics were predefined and listed in the research protocol. Collected data details are outlined in Table S3 in Multimedia Appendix 1. Results were aggregated into a table, organized with 1 column per database and 1 row per variable.

A descriptive analysis was then carried out on the gathered data using R (version 4.1.2; R Foundation for Statistical Computing) [26]. Categorical variables were summarized as counts and frequencies per category, while quantitative variables were expressed as medians and interquartile ranges. Bar charts were used to visualize the distribution of categorical variables and histograms and density curves for the quantitative variables. In addition, the temporal evolution of the studied variables was analyzed.

## Results

### Study Selection

**Figure 1:**
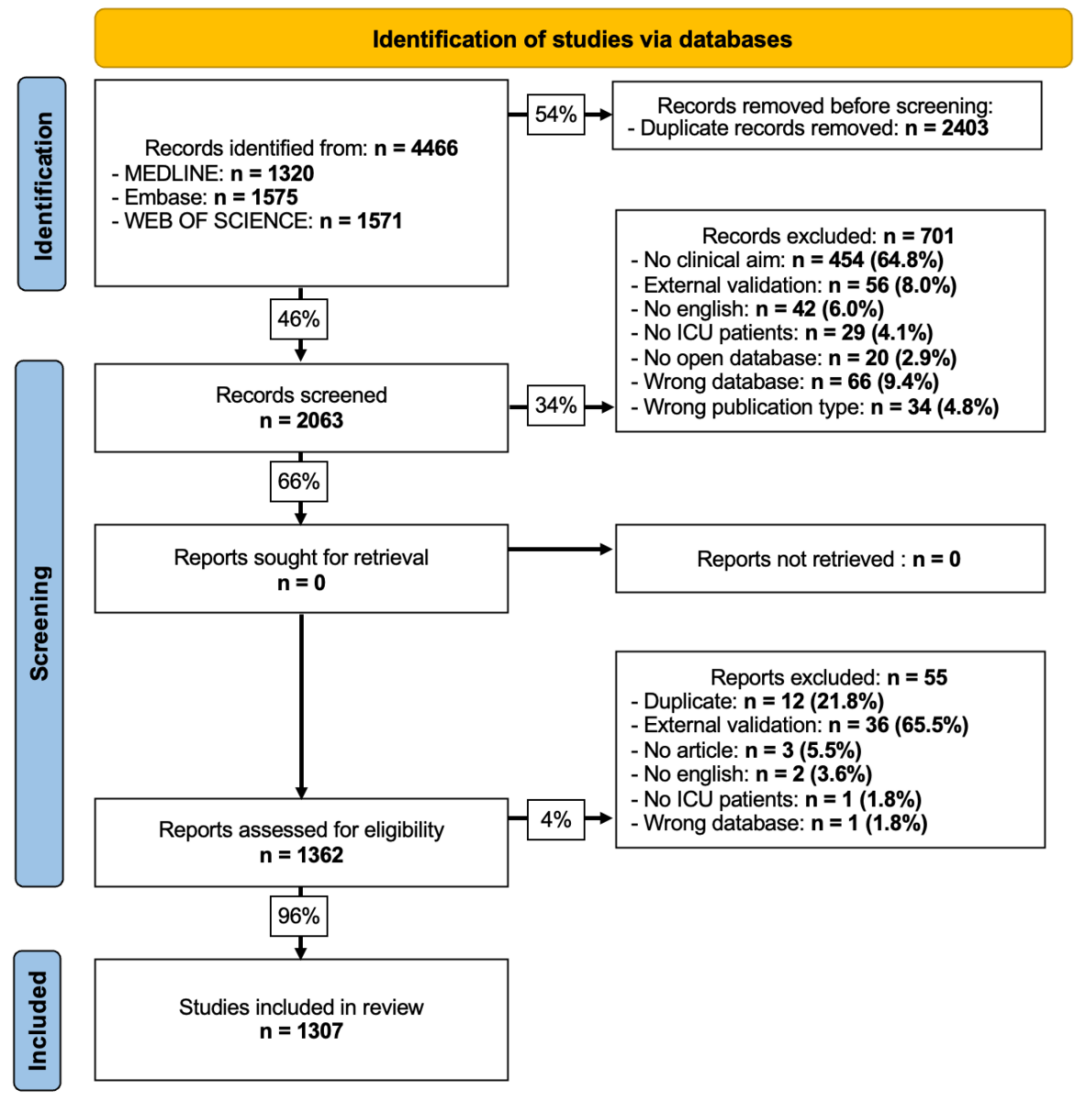
Flowchart of scoping review. ICU: intensive care unit.

From the inception of these databases up to August 2022, a total of 4466 publications were identified. After excluding duplicates, 2063 (46.2%) titles and abstracts were retained. Among these articles, 1362 (66%) met the inclusion criteria. Ultimately, 1307 (96%) articles were selected for the final analysis after thorough reading. The selection flowchart is shown in Figure 1, with the characteristics of the included studies are detailed in Table 1.

**Table 1:** Characteristics of studies.

Characteristics				Studies (N=1307)
**Article information**				
	**Date of publication, (year), n (%)**			
		2000-2010	6 (0.5)	
		2011-2015	38 (2.9)	
		2016-2020	296 (2.6)	
		2020-August 2022	967 (74.0)	
	**Database used, n (%)**			
		AmsterdamUMCdb^a^	6 (0.5)	
		eICU-CRD^b^	196 (15.0)	
		HiRID^c^	0 (0)	
		MIMIC^d^ II clinical database	107 (0.2)	
		MIMIC^d^ III clinical database	905 (9.2)	
		MIMIC^d^ IV clinical database	197 (15.1)	
**Number of databases used, n (%)**				
	1		1186 (91.5)	
	2		105 (0.1)	
	3		5 (0.4)	
Corresponding author’s sex, female, n (%)				352 (26.9)
**Country of corresponding author (top 10), n (%)**				
	China		689 (2.7)	
	United States		370 (28.3)	
	United Kingdom		38 (3)	
	Canada		17 (1.3)	
	Germany		16 (1.2)	
	Japan		13 (1.0)	
	India		11 (0.8)	
	Australia		10 (0.8)	
	Singapore		10 (0.8)	
	South Korea		10 (0.8)	
	Spain		10 (0.8)	
	Other		112 (8.5)	
**Journal information**				
	**Journal name (top 10), n (%)**			
		*Frontiers in Medicine*	52 (4.0)	
		*Critical Care Medicine*	41 (3.1)	
		*American Journal of Respiratory and Critical Care Medicine*	40 (3.1)	
		*Frontiers in Cardiovascular Medicine*	37 (2.8)	
		*International Journal of General Medicine*	36 (2.8)	
		*Intensive Care Medicine Experimental*	34 (2.6)	
		*Scientific Reports*	31 (2.4)	
		*Critical Care*	26 (2.0)	
		*Plos One*	25 (1.9)	
		*Chest*	24 (1.8)	
	Impact factor, median (IQR)		3.8 (2.8-5.8)	
**Study information, median (IQR)**				
	Inclusion period (years)		11 (11-11)	
	Number of participants		4282 (1468-12,740)	

^a^AmsterdamUMCdb: Amsterdam University Medical Centers Database.

^b^eICU-CRD: eICU Collaborative Research Database.

^c^HiRID: High time-resolution ICU dataset.

^d^MIMIC: Medical Information Mart for Intensive Care.

### Article Information

**Figure 2:**
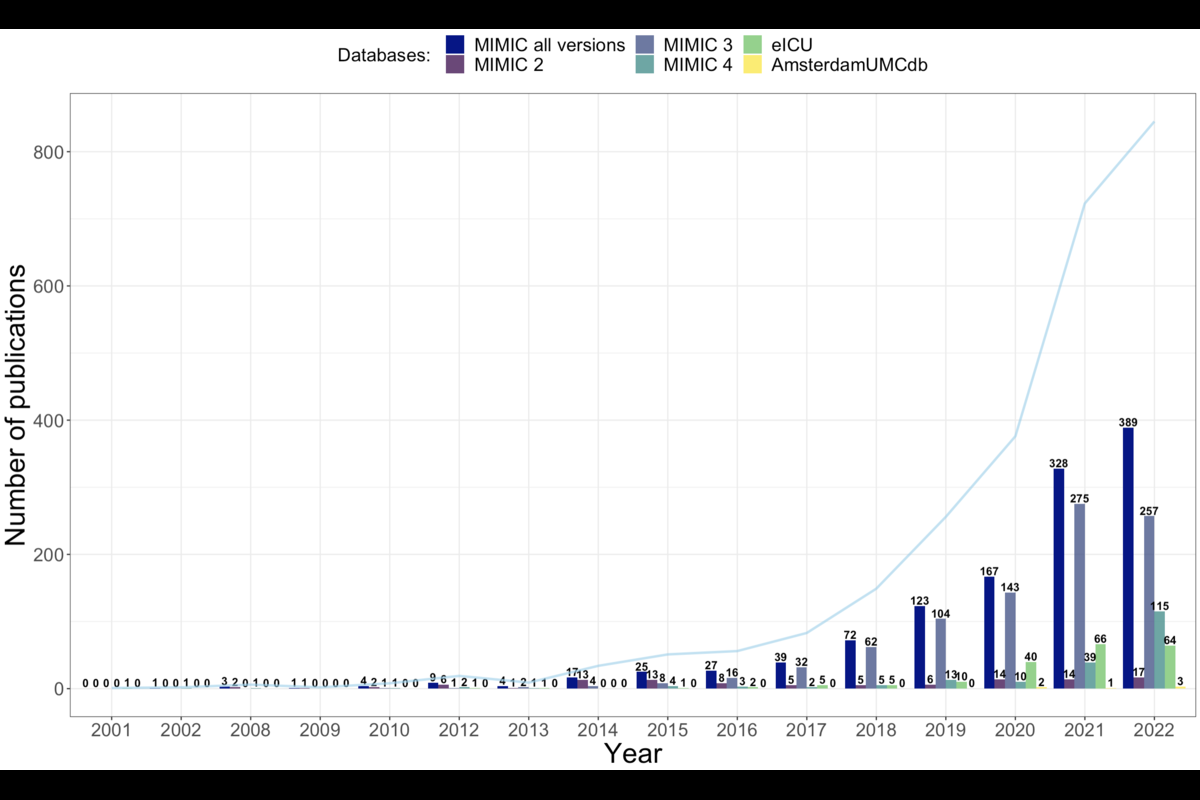
Evolution of the number of publications over the years. Blue line represents the total number of publications. AmsterdamUMCdb: Amsterdam University Medical Centers Database; eICU-CRD: eICU Collaborative Research Database; MIMIC: Medical Information Mart for Intensive Care.

**Figure 3:**
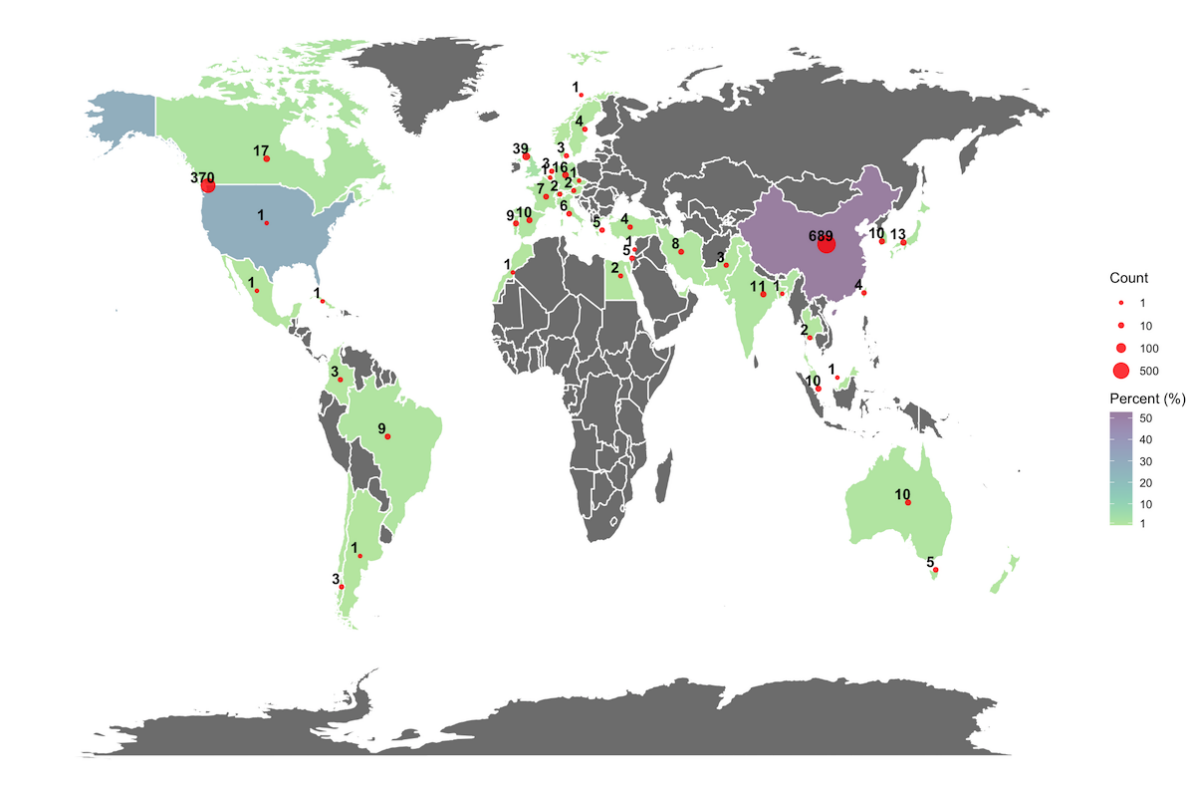
Worldwide publications number distribution. Red dots represent the number of publications per country. Country colors indicate the percentage of publications for each country.

Since 2016, there was a consistent rise in the number of publications using these databases. Most studies were published after the year 2020, with 967 (74.0%) publications. The MIMIC databases were the most frequently used. No publications using the HiRID database were identified (Table 1, Figure 2). China and the United States were the leading countries in terms of number of publications, with 689 (52.7%) and 370 (28.3%) articles, respectively. European countries contributed to 109 (8.3%) publications, led by the United Kingdom leading with 39 (3.0%) publications (Table 1, Figure 3).

### Journal Information

The median impact factor of publications was 3.8 (IQR 2.8-5.8) (Table 1). As for the journal field, the most commonly represented journals were those in medicine, computer science, and intensive care, with 712 (54.5%), 373 (28.5%), and 242 (18.5%) publications, respectively (Table 2).

**Table 2:** Field of the journal.

Field of the journal	Studies (N=1307), n (%)
Medicine	712 (54.5)
Informatics–mathematics–physics	373 (28.5)
Critical care	242 (18.5)
Biology–microbiology–immunology	82 (6.3)
Multidisciplinary sciences	63 (4.8)
Health care sciences–services	56 (4.3)
Pharmacology–pharmacy	39 (3)
Surgery	34 (2.6)
Environmental sciences	22 (1.7)
Neurosciences	22 (1.7)
Anesthesiology	21 (1.6)
Emergency	17 (1.3)
Genetics–heredity	12 (0.9)
Nursing	10 (0.8)
Management sciences	4 (0.3)

### Study Information

Studies included a median number of 4282 (IQR 1468-12,740) patients with a maximum sample size reaching up to 219,306 by combining patients from several databases. The median inclusion period was 11 (IQR 11-11) years (Table 1).

Research predominantly explored the cardiovascular system, particularly hemodynamics with 333 (25.5%) publications and infectious diseases, specifically sepsis with 324 (24.8%) publications. Renal failure and metabolic disorders were also heavily studied, with 231 (17.7%) publications, along with respiratory failure and mechanical ventilation, which had 203 (15.5%) publications. Notably, many studies investigated overall mortality among critically ill patients admitted to the ICU, with 178 (13.6%) publications (Table 3).

Furthermore, 422 (32.6%) studies investigated the entire population admitted to the ICU. Most studies focused on patients experiencing specific organ failures, represented in 818 (62.6%) publications (Table 4). Various exposure or prediction factors were evaluated. Biological data and vital signs were the most analyzed, featuring in 673 (51.5%) and 428 (32.7%) studies, respectively. Demographic information, medication prescriptions, and comorbidities were also widely included, with 332 (25.4%), 278 (21.3%) and 271 (20.7%) publications, respectively (Table 5). The most frequently assessed primary outcome was mortality, observed either within the ICU, the hospital, or after a certain period of time, across 878 (67.2%) studies (Table 6).

**Table 3:** Research topics.

Research topics	Studies (N=1307), n (%)
Cardiovascular–hemodynamics	333 (25.5)
Infectiology–immunology	324 (24.8)
Nephrology–urology–metabolic	231 (17.7)
Pulmonary–intubation–ventilation	203 (15.5)
General	178 (13.6)
Pharmacology	105 (8)
Endocrinology–nutrition	92 (7)
Neurology–neurosurgery	86 (6.6)
Digestive–hepatology	83 (6.4)
Hemostasis–thrombosis–transfusion	82 (6.3)
Traumatology–orthopedics	34 (2.6)
Sedation–curarization–analgesia	26 (2)
Technology–monitoring	25 (1.9)
Cancerology	24 (1.8)
Psychiatry	16 (1.2)
Geriatric	15 (1.1)
Sociology	15 (1.1)
Toxicology–addictology	13 (1)
Pediatric	12 (0.9)
Other	7 (0.5)
Ethics	1 (0.1)
Obstetrics	1 (0.1)

**Table 4:** Analyzed population.

Analyzed population	Studies (N=1307), n (%)
Patients with a specific symptom, disease, organ failure	818 (62.6)
All intensive care unit patients	428 (32.7)
Patients with a specific medical procedure	127 (9.7)
Patients after a surgical procedure	52 (4)
Patients with a specific biological disorder	6 (0.5)

**Table 5:** Analyzed exposures.

Analyzed exposures	Studies (N=1307), n (%)
Biological markers	673 (51.5)
Vital signs	428 (32.7)
Demographic characteristics	332 (25.4)
Treatments	278 (21.3)
Comorbidities	271 (20.7)
Scores	125 (9.6)
Morphological measurement	114 (8.7)
Ventilation settings	103 (7.9)
Social determinants	88 (6.7)
Language	85 (6.5)
Other	83 (6.4)
Mechanical ventilation	76 (5.8)
Type of admission	53 (4.1)
Diagnostic procedure	49 (3.7)
Length of stay	45 (3.4)
Type of intensive care unit	43 (3.3)
Fluid balance	40 (3.1)
Renal replacement therapy	35 (2.7)
Transfusion	20 (1.5)
Surgery	20 (1.5)
Cluster	18 (1.4)
Substance use disorder	16 (1.2)
Artificial nutrition	5 (0.4)

**Table 6:** Analyzed outcomes.

Analyzed outcomes	Studies (N=1307), n (%)
Mortality	878 (67.2)
Occurrence of a clinical event	327 (25)
Resource management	135 (10.3)
Biological markers	69 (5.3)
Vital signs	66 (5)
ICU^a^ readmission	35 (2.7)
Length of stay	27 (2.1)
Other	2 (0.2)

^a^ICU: intensive care unit.

### Statistical Methods Used and Results

Regarding the study objectives, 713 (54.6%) studies addressed an inference question, while 594 (45.4%) studies targeted a prediction question (Table 7, Table 8). Supervised learning and deep learning methods were the most commonly applied machine learning techniques, with 432 (33.1%) and 247 (18.9%) publications respectively (Table 7).

When examining specific models, logistic or linear regression was the predominant choice for inference questions with 396 (55.5%) publications (Table 9). Regarding prediction questions, logistic or linear regression was the most widely used classical machine learning method, surpassing survival models and generalized models with 281 (47.5%), 29 (4.9%), and 12 (2%) publications respectively. Neural networks were the most frequently applied advanced machine learning methods outpacing tree-based methods and boosting methods, with 237 (40.0%), 186 (31.4%), and 169 (28.5%) publications respectively (Table 10).

Inference studies reported statistically significant results in 84% of cases. Furthermore, the prevailing performance measure in prediction studies was the area under the curve (AUC) with 436 (73.4%) studies. The median AUC value was 0.840 (IQR 0.780-0.890; Table 8).

Preexisting clinical scores were applied for comparison with the performance of prediction models implemented in the studies. The most used scores were SAPS (Simplified Acute Physiology Score), SOFA (Sepsis-related Organ Failure Assessment), and APACHE (Acute Physiology and Chronic Health Evaluation) scores, with 55 (42.6%), 24 (18.6%), and 14 (10.9%) publications, respectively. The median AUC for these scores was 0.733 (IQR 0.677-0.781; Table 8).

**Table 7:** Algorithm used.

Algorithm used	Studies (N=1307), n (%)
Inference	713 (54.6)
Supervised learning	432 (33.1)
Deep learning	247 (18.9)
Unsupervised learning	39 (3)
Reinforcement learning	22 (1.7)

**Table 8:** Statistical analyses.

Characteristics and statistical methods used						Studies (N=1307)
**Aim of the study, n (%)**						
			Inference		713 (54.6)	
			Prediction		594 (45.4)	
**Effect-size measure (if inference), n (%)**						
			Odds ratio		348 (57.4)	
			Hazard ratio		244 (40.3)	
			Coefficient		14 (2.3)	
**Prediction performance measure (if prediction), n (%)**						
			Area under the receiving operating curve		436 (73.4)	
			Sensibility or specificity		143 (24)	
			Accuracy		142 (23.9)	
			*F*_1_-score		95 (16)	
			C-Index		74 (12.5)	
			Recall		42 (7.1)	
			Root-mean-square error		8 (1.3)	
**Known prediction scores used for comparison (if prediction), n (%)**						
			Simplified acute physiology score		55 (42.6)	
			Sepsis-related organ failure assessment		24 (18.6)	
			Acute physiology and chronic health evaluation		14 (10.9)	
			Acute physiology score		9 (7)	
			Modified early warning score		4 (3.1)	
			Oxford acute severity of illness		3 (2.3)	
			Other (<3 publications)		20 (15.5)	
**Key findings obtained**						
	**Effect-size value (Odds ratio or hazard ratio), mean (IQR)**					
		Protective effect		0.65 (0.50-0.79)		
		Adverse effect		1.63 (1.28-2.36)		
	***P*** **value, n (%)**					
		>.05		98 (16.)		
		.01-.05		151 (24.6)		
		.001-.01		95 (15.5)		
		<.001		269 (43.9)		
	Performance of models used (area under the curve), median (IQR)				0.840 (IQR 0.780-0.890)	
	Performance of known scores used (area under the curve), median (IQR)				0.733 (IQR 0.677-0.781)	

**Table 9:** Specific model used according to the aim of study (if inference).

Specific model used according aim of study (if inference)	Studies (N=713), n (%)
Linear or logistic regression	396 (55.5)
Survival regression	250 (35.1)
Propensity score matching	140 (19.6)
Descriptive–univariate	83 (11.6)
Other	48 (6.7)
Generalized additive model	24 (3.4)
Clustering	20 (2.8)
Natural language processing	2 (0.3)

**Table 10:** Specific model used according to the aim of study (if prediction).

Specific model used according aim of study (if prediction)	Studies (N=594), n (%)
Linear or logistic regression	282 (47.5)
Neural network	237 (39.9)
Decision tree or random forest	188 (31.6)
Boosting	171 (28.8)
Support vector machine	105 (17.7)
Natural language processing	81 (13.6)
Regularization (Lasso, Ridge, or Elasticnet)	78 (13.1)
K-nearest neighbors	49 (8.2)
Naive bayes	48 (8.1)
Other	40 (6.7)
Survival regression	29 (4.9)
Reinforcement learning	22 (3.7)
Clustering	19 (3.2)
Generalized additive model	12 (2.0)
Bagging	12 (2.0)
Bayesian network	7 (1.2)

## Discussion

### Principal Findings

This review examined clinical publications from open databases in the field of intensive care. We observed a consistent increase in the number of publications from these databases since 2016, with the majority of articles being published after 2020. MIMIC databases were the most frequently used, while the countries contributing the most were China and the United States. Most studies were published in journals with an impact factor ranging from 3 to 6. Cardiovascular and infectious topics, particularly those related to hemodynamics and sepsis, were the most represented. Other significant subjects included renal failure coupled with metabolic disorders and respiratory failure with mechanical ventilation. The most studied outcome measure was mortality in the ICU. Biological data, vital signs, demographic details, medication prescriptions, and comorbidities were extensively used as exposure or predictor factors.

Regarding statistical methods, logistic regression was the most used model for both inference and prediction questions. Neural networks were the most frequently used advanced machine learning methods surpassing supervised and reinforcement methods. A majority of the inference studies presented statistically significant results. In prediction studies, the most recurrent performance measure was the AUC, with a median value of 0.840.

### Comparison With Previous Work

The rising number of publications using open databases is steadily increasing, highlighting the potential impact of these resources on clinical research within intensive care. Collaborative initiatives have led to the emergence of large open-access databases, enhancing data availability in this field. Researchers have now access to data from numerous centers, enabling the production of robust results through larger sample sizes. Furthermore, this access has significantly boosted statistical power, enabling the study of specific topics and populations that were previously overlooked due to insufficient sample sizes. In our review, the median sample size in the studies was 4282 (IQR 1468-12,740), in contrast to typical sample sizes in prospective studies among critical care patients, that barely reach a few hundred patients. In a literature review by van de Sande et al [27] studying machine learning in intensive care, the median sample size was 179 (IQR 94-1411) and 142 (IQR 40-380) across all prospective observational and clinical studies, respectively.

The wealth and diversity of information contained in these databases have opened a broad spectrum of research possibilities, resulting in a wide array of topics covered in publications. Our review categorized these into over 20 different categories. Cardiovascular, infectious, respiratory, and metabolic issues emerged as the most commonly studied research domains, mirroring the reality of clinical practice in intensive care [28]. Mortality in ICU was the most frequently analyzed outcome measure. In a literature review by Syed et al [14], which examined the application of machine learning using the MIMIC dataset, mortality prediction was also the most studied outcome measure, followed by sepsis prediction, cardiac events, and acute kidney injury prediction.

Despite the significant number and diversity of publications, the clinical relevance of studies from these databases remains heterogeneous. The results were often statistically significant but had limited effect sizes. Nevertheless, there are few criteria to objectively evaluate the clinical relevance of a publication. In addition, the quantitative nature of our analyses did not allow us to fully explore this aspect. A qualitative methodology specifically assessing clinical relevance would provide more insights and discussion points.

Recently, a bibliometric analysis study using machine learning and natural language processing methods Bidirectional Encoder Representations from Transformers (BERTopic) compared research topics in traditional intensive care unit studies and those conducted with open access databases. A total of 1307studies from open access databases were identified versus 145,426 traditional studies. Among these 1307 studies, the most predominant topic was sepsis and kidney injury, and over 40% of the articles were based on predictive models [29].

### Toward Precision Intensive Care Medicine

In recent years, due to advancing technology, data availability, and the need to analyze increasingly larger databases, machine learning has emerged to develop increasingly accurate predictive models. Machine learning is a field of computer science and a part of artificial intelligence that defines both the science and engineering for which computer systems can analyze data and learn from the information contained within [30].

In our study, 594 out of 1307 (45.4%) publications used at least 1 machine learning model, with a significant increase in their implementation after 2015. The most commonly used machine learning method was logistic regression and neural networks. Literature review by Shillan et al [31], summarizing the characteristics and results of machine learning methods used in intensive care, found similar results. Nearly half of the studies identified in that review were published after 2015, and the most commonly used methods were neural networks, support vector machines, and decision trees.

Although the use of machine learning models in intensive care has increased in the scientific literature, their adoption in actual clinical practice remains limited or even absent at present. Machine learning models are often complex and require specialized skills to develop and implement. Their interpretation also poses a significant barrier to their use, with the phenomenon known as the “black box” issue [32]. Furthermore, these models need to be rigorously validated to ensure their reliability and accuracy. External validation of models on independent datasets is crucial to evaluate their performance under real-world conditions. In our review, the vast majority of publications analyzed came from databases implemented in the United States of America, limiting the generalizability of these models to the rest of the population. In addition, only 92 out of 2063 (4.5%) articles focused on external validation using an independent dataset, which is consistent with Shillan et al findings [31]. Furthermore, prospective clinical evaluation of these models is still rare. In a recent review, only 10 out of 494 (2%) articles clinically evaluated artificial intelligence in real clinical settings, with 5 studies being randomized clinical trials [27]. Finally, the use of machine learning models raises ethical and regulatory questions, particularly concerning data privacy, automated decision-making, and liability in case of errors. Clear guidelines and regulations must be established to govern the use of these models in a clinical context.

### Strengths and Limitations

This study, to our knowledge, is the first to examine the contribution of open databases in clinical research in intensive care. This study follows a rigorous methodology, and its research protocol was made public before the study [33]. Furthermore, this review adopts a comprehensive approach by including all clinical research publications generated from open ICU databases. By examining a wide range of publications, it provides a detailed overview of research themes, methodologies used, and results obtained. This can assist clinicians and researchers in understanding how these databases can contribute to improving patient care in intensive care and offering a solid foundation for future research and discussions.

However, it is important to acknowledge the limitations of this literature review. First, only publications written in English were included, potentially introducing a linguistic bias. Second, there exists and inherent risk of publication bias in literature reviews. Published and accessible studies may not represent all research carried out on open databases in intensive care. Studies with negative or nonsignificant results are less likely to be published, potentially resulting in an overestimation of positive results. While several open ICU databases were included in the study, it is possible that there exist other databases that were not taken into account. This could limit the representativeness of the sampled studies. Finally, despite exhaustive research, no publications from the HiRID database were identified. With these limitations in mind, it is important to consider the results of this study with caution and interpret them in the appropriate context.

### Conclusion

Open databases in intensive care have facilitated clinical research and provided new perspectives for enhancing care in intensive care. The abundance of scientific outputs resulting from these databases and the diversity of topics addressed highlight the importance of these databases as valuable resources for clinical research and suggest their potential impact on clinical practice in intensive care. However, the quality of studies and their clinical relevance remain highly heterogeneous among publications and are challenging elements to evaluate.

## Appendix

Multimedia Appendix 1 Additional material.

Multimedia Appendix 2 PRISMA-ScR Checklist.

## Abbreviations

AmsterdamUMCdb: Amsterdam University Medical Centers Database
APACHE: Acute Physiology and Chronic Health Evaluation
AUC: area under the curve
BERTopic: Bidirectional Encoder Representations from Transformers
eICU-CRD: eICU Collaborative Research Database
HiRID: High time-resolution ICU dataset
ICU: intensive care unit
MIMIC: Medical Information Mart for Intensive Care
PRISMA-ScR: Preferred Reporting Items for Systematic Reviews and Meta-Analyses extension for Scoping Reviews
SAPS: Simplified Acute Physiology Score
SOFA: Sepsis-related Organ Failure Assessment
