# Supplementary material for: Contribution of Open Access Databases to Intensive Care Medicine Research: Scoping Review
Source: J Med Internet Res. 2025 Jan 9;27:e57263. doi: 10.2196/57263 (PMC11757948; doi:10.2196/57263)
Supplement: Multimedia Appendix 1 [file jmir_v27i1e57263_app1.doc]

**Contribution of open access databases to intensive care medicine research:**

**a scoping review**

Julien Kallout^1^, MD MSc; Antoine Lamer^2^, PhD; Julien Grosjean^3^, PhD; Gaétan Kerdelhué^3^; Guillaume Bouzillé, MD PhD^4^; Thomas Clavier, MD PhD^1, 5^; Benjamin Popoff^1, 4^, MD MSc;

^1^CHU Rouen, Department of Anesthesiology and Critical Care, F-76000 Rouen, France

^2^Univ. Lille, CHU Lille, ULR 2694 - METRICS: Évaluation des Technologies de Santé et des Pratiques Médicales, Lille, France - Fédération Régionale de Recherche en Santé Mentale et Psychiatrie des Hauts-de-France, F-59350 Saint-André-lez-Lille, France.

^3^CHU Rouen, Department of Biomedical Informatics, F-76000 Rouen, France.

^4^CHU Rennes, INSERM, LTSI-UMR 1099, Univ Rennes, F-35000 Rennes, France

^5^Normandie Univ, UNIROUEN, INSERM U1096, Rouen, France.

**Supplementary materials**

- Table S1. Preferred Reporting Items for Systematic reviews and Meta-Analyses extension for Scoping Reviews (PRISMA-ScR) Checklist.
- Table S2. Search terms used for studies selection.
- Table S3. Collected variables.

**Table S1. Preferred Reporting Items for Systematic reviews and Meta-Analyses extension for Scoping Reviews (PRISMA-ScR) Checklist.**

| **Section**  **and Topic** | **Item** | **PRISAM-ScR checklist item** | **Reported**  **on page number** |
| --- | --- | --- | --- |
| **TITLE** | | | |
| Title | 1 | Identify the report as a scoping review. | 1 |
| **ABSTRACT** | | | |
| Structured summary | 2 | Provide a structured summary that includes (as applicable): background, objectives, eligibility criteria, sources of evidence, charting methods, results, and conclusions that relate to the review questions and objectives. | 2 |
| **INTRODUCTION** | | | |
| Rationale | 3 | Describe the rationale for the review in the context of what is already known. Explain why the review questions/objectives lend themselves to a scoping review approach. | 3 |
| Objectives | 4 | Provide an explicit statement of the questions and objectives being addressed with reference to their key elements or other relevant key elements used to conceptualize the review questions and/or objectives | 3 |
| **METHODS** | | | |
| Protocol and registration | 5 | Indicate whether a review protocol exists; state if and where it can be accessed and if available, provide registration information, including the registration number. | 4 |
| Eligibility criteria | 6 | Specify characteristics of the sources of evidence used as eligibility criteria and provide a rationale. | 4 |
| Information sources | 7 | Describe all information sources in the search, as well as the date the most recent search was executed. | 5 |
| Selection of sources of evidence | 8 | State the process for selecting sources of evidence included in the scoping review. | 5 |
| Data charting process | 9 | Describe the methods of charting data from the included sources of evidence and any processes for obtaining and confirming data from investigators | 5 |
| Data collection process | 10 | Specify the methods used to collect data from reports, including how many reviewers collected data from each report, whether they worked independently, any processes for obtaining or confirming data from study investigators, and if applicable, details of automation tools used in the process. | 5 |
| Data items | 11 | List and define all variables for which data were sought and any assumptions and simplifications made. | 24-26 |
| Critical appraisal of individual sources of evidence | 12 | If done, provide a rationale for conducting a critical appraisal of included sources of evidence; describe the methods used and how this information was used in any data synthesis. | 5 |

| **RESULTS** | | | |
| --- | --- | --- | --- |
| Selection of sources of evidence | 14 | Give numbers of sources of evidence screened, assessed for eligibility, and included in the review, with reasons for exclusions at each stage, ideally using a flow diagram. | 6 |
| Characteristics of sources of evidence | 15 | For each source of evidence, present characteristics for which data were charted and provide the citations | 7 |
| Critical appraisal within sources of evidence | 16 | If done, present data on critical appraisal of included sources of evidence (see item 12). | 8-15 |
| Results of individual sources of evidence | 17 | For each included source of evidence, present the relevant data that were charted that relate to the review questions and objectives. | 8-15 |
| Synthesis of results | 18 | Summarize and/or present the charting results as they relate to the review questions and objectives. | 8-15 |
| **DISCUSSION** | | | |
| Summary of evidence | 19 | Summarize the main results (including an overview of concepts, themes, and types of evidence available), link to the review questions and objectives, and consider the relevance to key groups. | 16-17 |
| Limitations | 20 | Discuss the limitations of the scoping review process. | 18 |
| Conclusions | 21 | Provide a general interpretation of the results with respect to the review questions and objectives, as well as potential implications and/or next steps. | 19 |
| **FUNDING** | | | |
| Funding | 22 | Describe sources of funding for the included sources of evidence, as well as sources of funding for the scoping review. Describe the role of the funders of the scoping review. | 22 |

**Table S2. Search terms used for studies selection.**

| **Database** | **Search terms** |
| --- | --- |
| **Pubmed** | "Medical Information Mart for Intensive Care"[Text word] OR "MIMIC database"[Text word] OR "MIMIC II"[Text word] OR "MIMIC III"[Text word] OR "MIMIC IV"[Text word] OR "Amsterdam University Medical Centers Database"[Text word] OR AmsterdamUMCdb[Text word] OR "eICU-CRD"[Text word] OR "eICU Collaborative Research Database"[Text word] OR "High time resolution ICU dataset"[Text word] OR HiRID[Text word] |
| **Embase** | TS=("Medical Information Mart for Intensive Care") OR TS=("MIMIC database") OR TS=("MIMIC II") OR TS=("MIMIC III") OR TS=("MIMIC IV") OR TS=("Amsterdam University Medical Centers Database") OR TS=(AmsterdamUMCdb) OR TS=(eICU-CRD) OR TS=("eICU Collaborative Research Database") OR TS=("High time resolution ICU dataset") OR TS=(HiRID) |
| **Web of science** | 'Medical Information Mart for Intensive Care' OR 'MIMIC database' OR 'MIMIC II' OR 'MIMIC III' OR 'MIMIC IV' OR 'Amsterdam University Medical Centers Database' OR AmsterdamUMCdb OR eICU-CRD OR 'eICU Collaborative Research Database' OR 'High time resolution ICU dataset' OR HiRID |

**Table S3. Collected variables.**

| **Variables** | **Definitions** |
| --- | --- |
| ***Article information*** |  |
| Title Digital |  |
| Digital Object Identifier (DOI) |  |
| Date of publication (YYYY) |  |
| Database used   - AmsterdamUMCdb - eICU-CRD - HiRID - MIMIC II Clinical database - MIMIC III Clinical database - MIMIC IV Clinical database |  |
| Corresponding author name |  |
| Corresponding author e-mail |  |
| Corresponding author gender |  |
| Corresponding author country |  |
| ***Journal information*** |  |
| Journal name |  |
| Field of the journal | Categories according to the Journal Citation Reports (JCR) **[26]**. |
| Impact factor |  |
| ***Study information*** |  |
| Inclusion period (years) | From the first inclusion of the first database to the last inclusion of the last database (if several databases). |
| Number of participants | Sums of patients included (from different databases if several databases). |
| Research topics   - General - Cardiovascular – Hemodynamics - Cancerology - Digestive – Hepatology - Endocrinology – Nutrition - Ethics - Geriatric - Hematology – Haemostasis – Transfusion - Infectiology – Immunology - Nephrology – Urology – Metabolic - Neurology – Neurosurgery - Obstetrics - Pediatric - Pharmacology - Psychiatry - Pulmonary – Intubation – Ventilation - Sedation – Curarisation – Analgesia - Technology – Monitoring - Toxicology – Addictology - Traumatology – Orthopedic - Other | Studies without specific theme (eg, mortality in critical patients).  Not belonging to the previous categories. |
| Population |  |
| - All intensive care unit patients | Publication studied the whole population of ICU patients. |
| - Patients with a specific symptom, disease or organ failure | Publication studied a specific population of ICU patients. |
| - Patients with a specific biological disorder |
| - Patients after a surgical procedure |
| - Patients with a specific medical procedure |
| - Other | Not belonging to the previous categories. |
| Exposure/Predictor |  |
| Demographic characteristics | Age, Gender. |
| Social determinants | The conditions in which people are born, grow, live, work and age. |
| Morphological measurement | Size and shape characters are quantified and reported as lengths or indices. |
| Substance use disorder | The persistent use of drugs despite substantial harm and adverse consequences as a result of their use. |
| Comorbidities | The simultaneous presence of two or more diseases or medical conditions in a patient. |
| Type of admission | Admission from the emergency department or for scheduled elective or emergency surgery. |
| Type of intensive care unit | Admission to a medical, surgical or specialized ICU. |
| Length of stay | Length of stay in ICU or hospital. |
| Vital signs | Clinical measurements that indicate the state of a patient's essential body functions. |
| Fluid balance | Measurement including intravenous fluids volume or output/input difference |
| Biological markers | Biological measures of a biological state evaluated as an indicator of normal biological processes, pathogenic processes or pharmacological responses to a intervention. |
| Diagnostic procedure | Test used to help diagnose a disease or condition other than laboratory tests. |
| Mechanical ventilation | Use of invasive or non-invasive mechanical ventilation. |
| Ventilation settings | The inputs to a mechanical ventilator that determine the mode and how much support is provided for the patient. |
| Renal replacement therapy | Use of a renal replacement method. |
| Artificial nutrition | Administration of enteral or parenteral nutrition. |
| Transfusion | Administration of labile blood products |
| Treatments | A set of measures applied to cure a disease, relieve symptoms, or prevent their onset including pharmacological and electrolytic treatments. |
| Surgery | Recourse to surgical intervention or type of surgical intervention. |
| Scores | Medical decision support tool, aggregating in a single value several clinical observations. |
| Language | Variables obtained from a natural language processing method. |
| Cluster | Groups obtained from an unsupervised learning method. |
| Other | Not belonging to the previous categories. |

| Outcome |  |
| --- | --- |
| Mortality | Explanation or prediction of mortality. |
| Occurrence of a clinical event | Explanation or prediction of a symptom, disease or organ failure. |
| Vital signs | Explanation or prediction of a vital signs. |
| Biological markers | Explanation or prediction of a biological markers. |
| Resource management | Explanation or prediction of therapeutic or management strategy. |
| ICU readmission | Explanation or prediction of discharge and readmission. |
| Length of stay | Explanation or prediction length of stay in ICU or hospital. |
| Other | Not belonging to the previous categories. |
| ***Statistical methods used and results*** |  |
| Aim of the study |  |
| Inference | Process of evaluating the relationship between the explanatory and response variables. |
| Prediction | Process of using a model to make a prediction about something that is yet to happen. |
| Algorithm used |  |
| Unsupervised learning | A machine learning method whose goal is to describe the associations and patterns among a set of input measures without outcome measure **[27]**. |
| Supervised learning | A machine learning method whose goal is to predict the value of an outcome measure based on input measures **[27]**. |
| Reinforcement learning | A machine learning method where an agent is faced an a problem and that learns behavior through trial-and-error interactions with a dynamic environment **[28]**. |
| Deep learning | A machine learning method usually performed by an artificial neural network composed of several layers of neurons arranged hierarchically and interacting with each other to predict the value of an outcome measure **[27]**. |
| Specific model used | Exhaustive list of used models. |
| Effect-size measure (if inference) | Type of measurement of effect size of primary outcome (if several). |
| Effect-size value (if inference) | Value of effect size of primary outcome (if several). |
| P-value (if inference) | P-value of primary outcome (if several). |
| Prediction performance measure (if prediction) | Type of measurement of performance of better model used (if several). |
| Prediction performance value (if prediction) | Value of performance of better model used (if several). |
